# Basophil-derived IL-4 promotes cutaneous Staphylococcus aureus infection

**DOI:** 10.1172/jci.insight.149953

**Published:** 2021-11-08

**Authors:** Juan-Manuel Leyva-Castillo, Mrinmoy Das, Jennifer Kane, Maria Strakosha, Sonal Singh, Daniel Sen Hoi Wong, Alexander R. Horswill, Hajime Karasuyama, Frank Brombacher, Lloyd S. Miller, Raif S. Geha

**Affiliations:** 1 Division of Immunology, Boston Children’s Hospital, Boston, Massachusetts, USA.; 2 Department of Pediatrics, Harvard Medical School, Boston, Massachusetts, USA.; 3 Department of Immunology and Microbiology, University of Colorado School of Medicine, Aurora, Colorado, USA.; 4 Inflammation, Infection and Immunity Laboratory, TMDU Advanced Research Institute, Tokyo Medical and Dental University (TMDU), Tokyo, Japan.; 5 International Center for Genetic Engineering and Biotechnology Cape Town Component and Health Science Faculty, University of Cape Town, Cape Town, South Africa.; 6 Department of Dermatology, Johns Hopkins University School of Medicine, Baltimore, Maryland, USA.

**Keywords:** Immunology, Infectious disease, Bacterial infections, Basophils, Innate immunity

## Abstract

Superficial cutaneous *Staphylococcus aureus* (*S*. *aureus*) infection in humans can lead to soft tissue infection, an important cause of morbidity and mortality. IL-17A production by skin TCR**γδ**^+^ cells in response to IL-1 and IL-23 produced by epithelial and immune cells is important for restraining *S*. *aureus* skin infection. How *S*. *aureus* evades this cutaneous innate immune response to establish infection is not clear. Here we show that mechanical injury of mouse skin by tape stripping predisposed mice to superficial skin infection with *S*. *aureus*. Topical application of *S*. *aureus* to tape-stripped skin caused cutaneous influx of basophils and increased *Il4* expression. This basophil-derived IL-4 inhibited cutaneous IL-17A production by TCR**γδ**^+^ cells and promoted *S*. *aureus* infection of tape-stripped skin. We demonstrate that IL-4 acted on multiple checkpoints that suppress the cutaneous IL-17A response. It reduced *Il1* and *Il23* expression by keratinocytes, inhibited IL-1+IL-23–driven IL-17A production by TCR**γδ**^+^ cells, and impaired IL-17A–driven induction of neutrophil-attracting chemokines by keratinocytes. IL-4 receptor blockade is shown to promote *Il17a* expression and enhance bacterial clearance in tape-stripped mouse skin exposed to *S*. *aureus*, suggesting that it could serve as a therapeutic approach to prevent skin and soft tissue infection.

## Graphical abstract

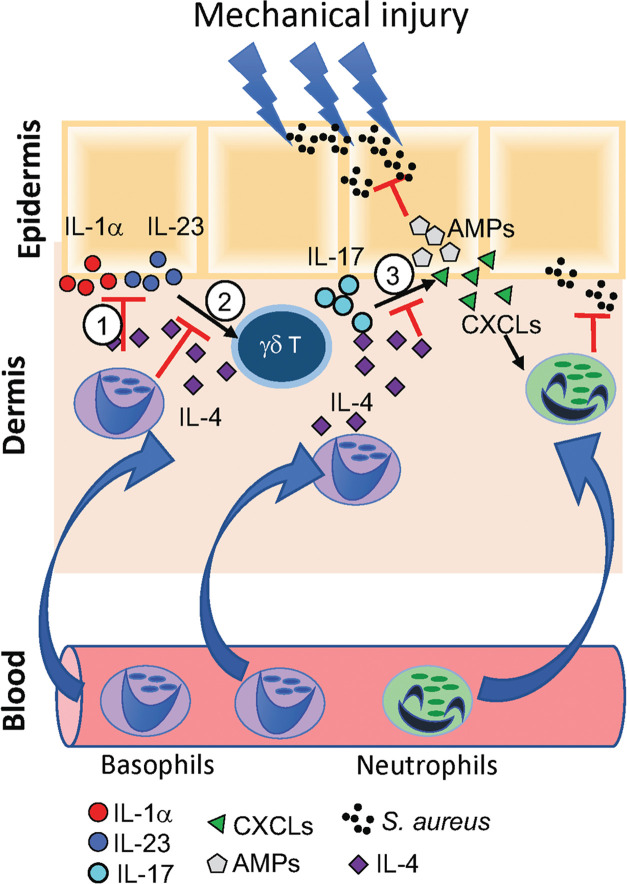

## Introduction

*Staphylococcus aureus (S. aureus*) is the most common causative agent of bacterial skin and soft tissue infections (SSTIs) (1–3). Impetigo, a superficial skin infection that commonly affects children, can lead to SSTIs. *S*. *aureus* commonly infects the skin of healthy individuals following trauma, burns, or surgical interventions, as well as the skin of patients with chronic diseases characterized by itching, such as atopic dermatitis (AD) and diabetes, suggesting that skin barrier disruption predisposes to cutaneous *S*. *aureus* infection (3–6).

IL-17A is a proinflammatory cytokine produced by CD4^+^ Th17 cells, TCRγδ^+^ cells, and type 3 innate lymphoid cells (ILC3s). IL-17A plays an important role in protection against mucosal infections, by recruiting neutrophils and promoting the production of antimicrobial peptides (AMPs) by epithelial cells (7, 8). Patients and mice with defects in IL-17A, IL-17A receptor chains, or IL-17A signaling, or defects in neutrophil number or function, are susceptible to mucosal and skin infections caused by *S*. *aureus* (9–11). In particular, IL-17A production by TCRγδ^+^ T cells and subsequent neutrophil recruitment to the skin play a critical role in controlling cutaneous *S*. *aureus* infection (7, 8, 11–14). The cytokines IL-1 and IL-23 are produced by several cells in the skin, including keratinocytes, DCs, and macrophages, and play an important role in driving IL-17A production (15, 16). Patients and mice with defects in IL-1 and IL-23 production or signaling have defective IL-17A production and are susceptible to *S*. *aureus* infections (16–22).

Basophils account for less than 1% of peripheral blood leukocytes. Basophils produce IL-4 and IL-13 and have been identified as important players in type 2 immune responses against allergens and parasitic infection (23). IL-4 has been shown to inhibit IL-17A production via STAT6 inhibition to the *Il17a* promoter (24). Basophils may promote Th17 responses by secreting IL-6 (25–28). Moreover, basophil-derived TNF-α enhances survival in sepsis induced by cecal ligation and puncture in mice (29). It is not known whether basophils and their products play a role in cutaneous *S*. *aureus* infection.

The mechanisms by which *S*. *aureus* circumvents the innate immune response to establish infection in injured skin are not well understood. We show that tape stripping, which only disrupts the upper layers of the epidermis, rendered mouse skin susceptible to *S*. *aureus* infection and that IL-4 derived from basophils recruited into *S*. *aureus*–exposed, tape-stripped skin inhibited cutaneous IL-17A production and promoted *S*. *aureus* skin infection. We further demonstrate that IL-4 inhibited *Il1b* and *Il23* expression in keratinocytes, acted on TCRγδ^+^ cells to inhibit their IL-17A production in response to IL-1β and IL-23, and blocked IL-17A–driven induction of neutrophil-attracting chemokines in keratinocytes. Furthermore, we show that IL-4 signaling blockade reversed the susceptibility of tape-stripped skin to *S*. *aureus* infection.

## Results

### Mechanical skin injury promotes superficial cutaneous infection by S. aureus.

**Figure 1:**
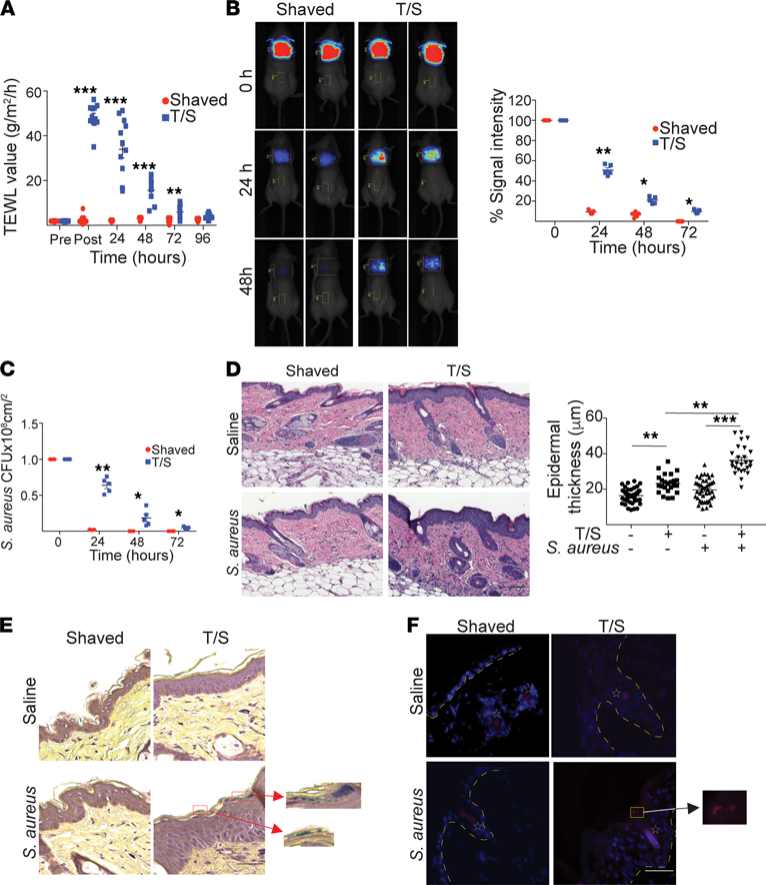
Mechanical skin injury promotes superficial cutaneous infection by *S. aureus*. (**A**) Effect of tape stripping on TEWL. (**B**) Representative in vivo fluorescence imaging (left) and quantitative analysis of the decay in PSVue 794 fluorescence (right) following topical application of 1 × 10^8^ PSVue 794–labeled *S*. *aureus* strain USASF8300 to shaved and non–tape-stripped or shaved and tape-stripped (T/S) skin of BALB/c mice. (**C**) Numbers of colony forming units (CFU) in skin homogenates following application of 1 × 10^8^ PSVue 794–labeled *S*. *aureus* strain USASF8300 to shaved or T/S skin of BALB/c mice. (**D**) Representative H&E staining (left) and quantitation of epidermal thickness of shaved or T/S skin sections from BALB/c mice 72 hours after application of saline or PSVue 794–labeled *S*. *aureus* strain USASF8300. Scale bar: 100 μm. (**E**) Gram staining of skin sections from the same experiment as in **D**. Red squares highlight Gram-positive bacteria. Scale bar: 50 μm. (**F**) Representative fluorescence images of sections of shaved or T/S skin of BALB/c mice 72 hours after application of saline or GFP-labeled *S*. *aureus* LAC strain. Yellow square highlights GFP^+^ bacteria; stars indicate autofluorescent hair follicles. Scale bar: 50 μm. Results in **A**–**D** are representative of 2 independent experiments with 4–5 mice/group. *P < 0.05, **P < 0.005, ***P < 0.001 by 1-tailed Student’s *t* test (**A**–**C**) or 1-way ANOVA (**D**).

We examined the clearance of topically applied *S*. *aureus* from shaved skin without or with tape stripping. Tape stripping resulted in a significant increase in transepidermal water loss (TEWL) that persisted for 72 hours (Figure 1A), demonstrating effective disruption of the skin barrier. A total of 1 × 10^8^ colony-forming units (CFU) of community-acquired methicillin-resistant *S*. *aureus* (MRSA; parental strain USA300 SF8300) labeled with the fluorescent dye PSVue 794 were applied topically. The SF8300 MRSA strain used in this study was the most prevalent *S*. *aureus* strain causing infections in the United States in the early 2000s, is one of the better characterized MRSA strains, and is the one most commonly used in laboratory studies (30–32). Persistence of *S*. *aureus* was determined at 0, 24, 48, and 72 hours by in vivo fluorescence imaging, as well as by measuring the numbers of CFU in skin homogenates. *S*. *aureus* was virtually completely cleared from shaved skin 24 hours after its application, as evidenced by the extinction of PSVue 794 fluorescence (Figure 1B) and by the recovery of negligible numbers of CFU at 24 hours and none at 48 and 72 hours (Figure 1C). In contrast, the decay in PSVue 794 fluorescence was significantly slower after application of *S*. *aureus* to tape-stripped skin, persisting throughout 48 hours (Figure 1B). Moreover, the numbers of CFU recovered at 24 hours were more than half those applied on day 0, and viable *S*. *aureus* bacteria were consistently recovered at 48 and 72 hours (Figure 1C).

Histologic examination of skin sections taken on day 3 revealed that tape stripping caused epidermal hyperplasia that was further increased by application of *S*. *aureus* (Figure 1D). Importantly, Gram staining revealed the presence of Gram-positive bacteria in the epidermis of tape-stripped, but not shaved, skin exposed to *S*. *aureus* (Figure 1E). Immunofluorescence examination on day 3 following topical application of GFP-expressing, community-acquired MRSA strain USA300 LAC revealed the presence of GFP^+^ bacteria in the epidermis of tape-stripped skin but not shaved skin (Figure 1F). Taken together, these results indicate that mechanical injury of skin promotes superficial cutaneous infection with *S*. *aureus*.

### IL-17A from TCRγδ^+^ cells restrains S. aureus infection of mechanically injured skin.

**Figure 2:**
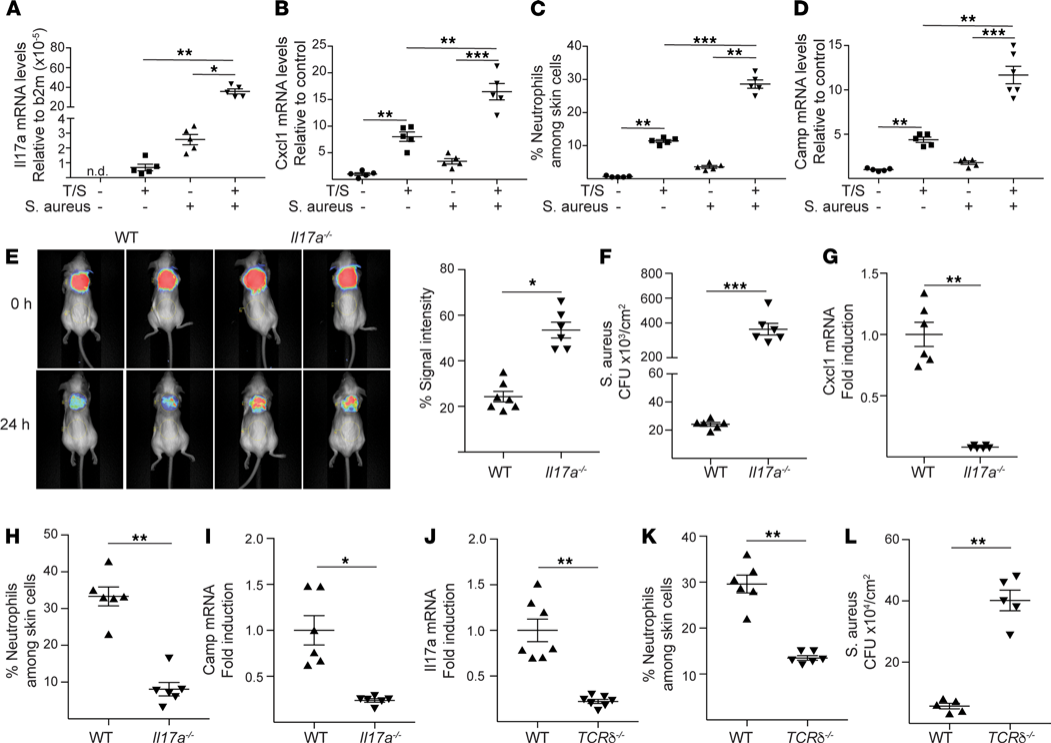
IL-17A from TCRγδ+ cells protects mechanically injured skin from superficial infection by *S. aureus*. (**A**–**D**) *Il17a* expression (**A**), *Cxcl1* expression (**B**), percentage of neutrophils (**C**), and *Camp* expression (**D**) in non–tape-stripped or tape-stripped (T/S) skin of BALB/c mice 24 hours after application of *S*. *aureus* (+) or saline (-) control. (**E**) Representative in vivo fluorescence imaging (left) and quantitative analysis of the PSVue 794 fluorescence 0 and 24 hours (right) following topical application of 1 × 10^8^ PSVue 794 labeled *S*. *aureus* strain USASF8300 to shaved and tape stripped (T/S) skin of *Il17a^–/–^* mice and WT controls. (**F**) Numbers of CFU in skin homogenates 72 hours following application of 1 × 10^8^ *S*. *aureus* strain USASF8300 to T/S skin of *Il17a^–/–^* mice and WT controls. (**G**–**I**) *Cxcl1* expression (**G**), percentage of neutrophils among skin cells (**H**), and *Camp* expression (**I**) in T/S skin of *Il17a^–/–^* mice and WT controls 24 hours following application of 1 × 10^8^ *S*. *aureus* to T/S skin of *Il17a^–/–^* mice and WT controls. (**J**–**L**) *Il17a* expression (**J**) and neutrophil infiltration (**K**) at 24 hours and numbers of CFU (**L**) at 72 hours in T/S skin from *Tcrd^–/–^* mice and WT controls following application of 1 × 10^8^ *S*. *aureus*. n.d., not detected. Results in **A**–**L** are representative of 2 independent experiments with 4–5 mice/group. *P < 0.05, **P < 0.005, ***P < 0.001 by 1-way ANOVA (**A**–**D**) or 1-tailed Student’s *t* test (**E**–**L**). b2m, beta-2-microglobulin.

IL-17A is important for the clearance of intradermally injected *S*. *aureus* (8, 14). We examined the IL-17A response to superficial skin infection with *S*. *aureus*. *Il17a* expression was not detectable in shaved mouse skin but was minimally induced by tape stripping (Figure 2A). *S*. *aureus* application modestly upregulated *Il17a* expression in shaved skin (Figure 2A). In contrast, it caused a drastic increase in *Il17a* expression in tape-stripped skin (Figure 2A). *S*. *aureus* application to shaved skin caused minimal or no increase in the expression of the neutrophil-attracting chemokines *Cxcl1*, *Cxcl2*, and *Cxcl3*; modest neutrophil infiltration; and no detectable increase in the expression of the AMP-encoding genes cathelicidin AMP (*Camp*) and defensin beta 14 (*Defb14*) (Figure 2, B–D; and Supplemental Figure 1, A and B; supplemental material available online with this article; https://doi.org/10.1172/jci.insight.149953DS1). *Cxcl1*, *Cxcl2*, and *Cxcl3* expression; neutrophil infiltration; as well as *Camp*, *Defb1*, *Defb3*, *Defb4*, and *Defb14* expression increased modestly after tape stripping but increased markedly after application of *S*. *aureus* to tape-stripped skin (Figure 2, B–D; and Supplemental Figure 1, A and B). These results indicate that *S*. *aureus* infection of mechanically injured skin causes a robust local IL-17A and AMP induction.

To investigate whether IL-17A restrains superficial skin infection by *S*. *aureus*, we examined *Il17a^–/–^* mice. Skin fluorescence 24 hours following application of PSVue 794-labeled *S*. *aureus* to tape-stripped skin was significantly higher in *Il17a^–/–^* mice compared with WT controls (Figure 2E). Moreover, the numbers of *S*. *aureus* CFU recovered from skin homogenates on day 3 were significantly higher in *Il17a^–/–^* mice compared with WT controls (Figure 2F). As expected, cutaneous expression of *Cxcl1*, *Cxcl2*, *Cxcl3*; neutrophil infiltration; and expression of *Camp*, *Defb4*, and *Defb14* after *S*. *aureus* application to tape-stripped skin were all significantly reduced in *Il17a^–/–^* mice compared with WT controls (Figure 2, G–I, and Supplemental Figure 1C).

TCRγδ^+^ cells, CD4^+^TCRαβ^+^ Th17 cells, and ILC3s produce IL-17A (7). *Tcrd^–/–^* mice, which lack TCRγδ^+^ cells, but have normal numbers of TCRαβ^+^ cells and ILC3s, failed to upregulate cutaneous *Il17a* expression and had decreased neutrophil influx and higher loads of *S*. *aureus* following *S*. *aureus* application to tape-stripped skin (Figure 2, J–L). In addition, intracellular flow cytometry demonstrated that TCRγδ^+^ cells but not CD4^+^ T cells or ILC3s isolated from *S*. *aureus*–infected skin were the main source of IL-17A produced after PMA and ionomycin stimulation (Supplemental Figure 2). These results indicate the importance of IL-17A produced by TCRγδ^+^ cells in clearing *S*. *aureus* infection from mechanically injured skin.

### IL-1 and IL-23 promote cutaneous Il17a expression in mechanically injured skin infected by S. aureus.

**Figure 3:**
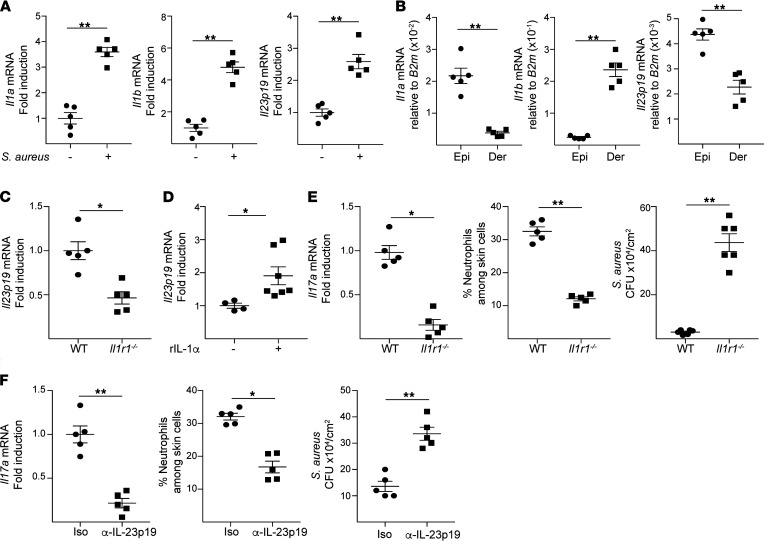
Epidermal cell–derived IL-1 and IL-23 promote cutaneous *Il17a* expression in mechanically injured skin infected by *S. aureus*. (**A** and **B**) *Il1a*, *Il1b*, and *Il23p19* expression in full-thickness skin (**A**) and epidermal and dermal sheets (**B**) 24 hours following application of 1 × 10^8^ *S*. *aureus* to tape-stripped (T/S) skin of BALB/c mice. (**C**) *Il23p19* expression in T/S skin 24 hours following topical application of 1 × 10^8^ *S*. *aureus* in *Il1r1^–/–^* mice and WT controls. (**D**) Induction of *Il23p19* expression in epidermal sheets of mouse skin by rIL-1α. (**E**) *Il17a* mRNA levels (left), percentages of neutrophils among skin cells (middle), and numbers of CFU (right) 72 hours following application of 1 × 10^8^ *S*. *aureus* to T/S skin of *Il1r1^–/–^* mice and WT controls. (**F**) *Il17a* mRNA levels (left), percentages of neutrophils among skin cells (middle), and numbers of CFU (right) 72 hours following application of 1 × 10^8^ *S*. *aureus* to T/S skin of WT BALB/c mice treated with anti–IL-23p19 antibody or isotype controls. Results in **A**–**F** are representative of 2 independent experiments with 4–5 mice/group. *P < 0.05, **P < 0.005 by 1-tailed Student’s *t* test.

The cytokines IL-1 and IL-23 drive IL-17A production by TCRγδ^+^ cells (16). Expression of *Il1a*, *Il1b*, and *Il23p19* in tape-stripped skin markedly increased following application of *S*. *aureus* (Figure 3A). Keratinocytes express predominantly *Il1a* while both keratinocytes and myeloid cells express *Il1b* and *Il23p19* (33–37). *Il1a* expression in tape-stripped skin exposed to *S*. *aureus* was confined predominantly to the epidermis. In contrast, *Il1b* was expressed predominantly in the dermis. *Il23p19* was expressed in both layers but significantly more in epidermis than dermis (Figure 3B). Upregulation of *Il23p19* expression in tape-stripped skin exposed to *S*. *aureus* was virtually abolished in *Il1r1*^–/–^ mice (Figure 3C). Moreover, recombinant IL-1α (rIL-1α) induced *Il23p19* expression in epidermal sheets from WT mice (Figure 3D), suggesting an autocrine loop was involved in IL-23 production by keratinocytes.

The role of IL-1 and IL-23 in inducing a protective IL-17A response in *S*. *aureus*–infected, mechanically injured skin was examined in *Il1r1*^–/–^ mice and following IL-23 blockade in WT mice, respectively. Cutaneous *Il17a* expression, neutrophil infiltration, and *S*. *aureus* clearance were significantly decreased in *Il1r1^–/–^* mice compared with WT controls as well as in mice treated with neutralizing anti–IL-23p19 antibody compared with mice treated with IgG isotype control (Figure 3, E and F). These results indicate that both IL-1 and IL-23 play an important role in driving IL-17A production by TCRγδ^+^ cells during superficial skin infection.

### Basophil-derived IL-4 suppresses cutaneous Il17a expression and promotes S. aureus infection of mechanically injured skin.

**Figure 4:**
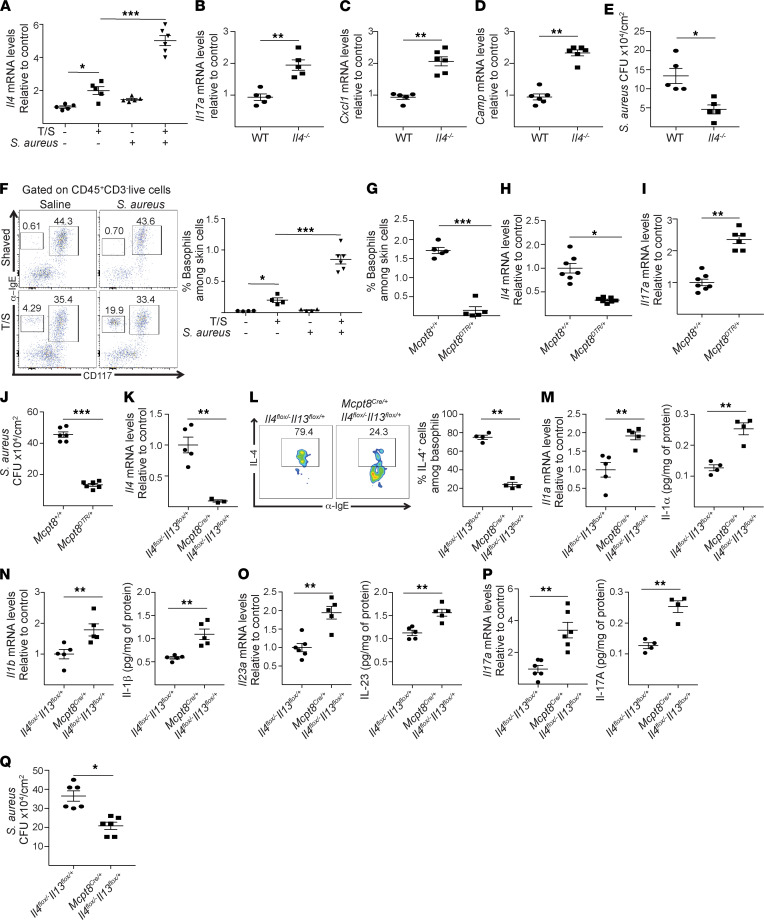
Basophil-derived IL-4 suppresses cutaneous *Il17a* expression and promotes *S. aureus* superficial infection of mechanically injured skin. (**A**) Cutaneous *Il4* expression 24 hours following topical application of 1 × 10^8^ *S*. *aureus* to shaved or shaved and tape-stripped (T/S) skin of BALB/c mice. (**B**–**E**) Cutaneous expression of *Il17a* (**B**), *Cxcl1* (**C**), and *Camp* (**D**) at 24 hours, and numbers of CFU (**E**), 72 hours following topical application of *S*. *aureus* to T/S skin from *Il4^–/–^* mice and WT controls. (**F**) Representative flow cytometry analysis of CD117 and FcεRI expression by CD45^+^CD3^–^ cells (left) and quantitation of CD45^+^CD3^–^FcεRI^+^CD117^–^ basophils (right) in shaved and shaved and T/S skin of BALB/c mice 24 hours after application of *S*. *aureus*. (**G**) Quantitation of CD45^+^CD3^–^FcεRI^+^CD117^–^ basophils in T/S skin of DT-treated *Mcpt8^DTR/+^* and *Mcpt8^+/+^* controls 24 hours after topical application of *S*. *aureus*. (**H**–**J**) *Il4* expression (**H**) and *Il17a* expression (**I**) at 24 hours and numbers of CFU (**J**) in T/S skin of DT-treated *Mcpt8^DTR/+^* and *Mcpt8^+/+^* controls 72 hours after topical application of *S*. *aureus*. (**K**–**P**) *Il4* expression (**K**), representative flow cytometry analysis of IL-4–producing basophils (left) and quantitation (right) (**L**), levels of *Il1a* mRNA (left) and IL-1α protein (right) (**M**), levels of *Il1b* mRNA (left) and IL-1α (right) (**N**), *Il23* mRNA (left) and IL-23 (right) levels (**O**), and levels of *Il17a* mRNA (left) and IL-17A (right) (**P**) at 24 hours, and numbers of *S*. *aureus* CFU (**Q**), 72 hours after application of *S*. *aureus* to T/S skin of *Mcpt8^Cre/+^* *Il4^fl/–^Il13^fl/+^* mice and *Il4^fl/–^Il13^fl/+^* controls. Results in **A**–**P** are representative of 2 independent experiments with 4–5 mice/group. *P < 0.05, **P < 0.005, ***P < 0.001 by 1-way ANOVA (**A** and **F**) or 1-tailed Student’s *t* test (**B**–**E** and **H**–**Q**).

IL-4 impairs the induction and maintenance of adaptive Th17 immune responses (36). We investigated whether IL-4 suppresses the TCRγδ^+^ cell–dependent cutaneous IL-17A response that protects tape-stripped skin from superficial infection with *S*. *aureus*. Expression of *Il4* was minimal in shaved skin and did not increase following application of *S*. *aureus* (Figure 4A). Tape stripping significantly increased *Il4* expression in the skin; exposure to *S*. *aureus* increased it further (Figure 4A). Cutaneous expression of *Il17a* and of the chemokine *Cxcl1* and of the AMP *Camp* following application of *S*. *aureus* to tape-stripped skin was significantly increased in *Il4^–/–^* mice compared with WT controls (Figure 4, B–D). Importantly, the numbers of *S*. *aureus* CFU recovered from skin homogenates after *S*. *aureus* application to tape-stripped skin were significantly decreased in *Il4^–/–^* mice compared with WT controls (Figure 4E). 

Basophils are an important source of innate cell-derived IL-4 (38). Flow cytometry analysis of skin cell suspensions revealed that CD45^+^CD3^–^IgE^+^CD117^–^ basophils accounted for less than 1% of CD45^+^CD3^–^ cells in shaved mouse skin before and after application of *S*. *aureus*. This percentage significantly increased 24 hours after tape stripping and markedly increased following application of *S*. *aureus* to tape-stripped skin (Figure 4F). In contrast, tape stripping and *S*. *aureus* application caused minimal changes in the percentages of IgE^+^CD117^+^ mast cells in the skin (Figure 4F). To investigate whether basophils are a major source of IL-4 in tape-stripped skin, *Mcpt8^DTR/+^* mice, which express diphtheria toxin receptor (DTR) selectively in basophils (39), and their *Mcpt8^+/+^* littermates were treated with DT. The percentage of basophils in tape-stripped skin exposed to *S*. *aureus* was drastically decreased in DT-injected *Mcpt8^DTR/+^* mice compared with DT-injected *Mcpt8^+/+^* controls (Figure 4G). Importantly, *Il4* expression in tape-stripped skin sites exposed to *S*. *aureus* was significantly diminished in DT-injected *Mcpt8^DTR/+^* mice compared with controls (Figure 4H). In contrast, *Il17a* expression at these sites was significantly higher in DT-injected *Mcpt8^DTR/+^* mice compared with controls (Figure 4I). Importantly, the numbers of CFU recovered from skin homogenates after *S*. *aureus* application to tape-stripped skin were significantly reduced in DT-injected *Mcpt8^DTR/+^* mice compared with controls (Figure 4J).

To investigate the role of basophil-derived IL-4 in *S*. *aureus* colonization of mechanically injured skin, we generated *Mcpt8^cre/+^ Il4^fl/–^13^fl+^* mice with selective ablation of IL-4 in basophils. To create this line, we crossed *Mcpt8^cre/+^* mice on IL-4–deficient background (*Mcpt8^cre/+^ Il4^–/–^* mice) with *Il4/13^fl/fl^* mice. *Mcpt8^cre/+^ Il4^fl/–^13^fl/+^* mice, but not *Il4^fl/+^13^fl/+^* control littermates, failed to upregulate cutaneous *Il4* expression after application of *S*. *aureus* to tape-stripped skin (Figure 4K). In addition, basophils from *Mcpt8^cre/+^ Il4^fl/–^13^fl/+^* mice were severely impaired in their ability to produce IL-4 compared with basophils from *Il4^fl/+^13^fl/+^* control littermates (Figure 4L). In contrast, cutaneous IL-1α, IL-1β, and IL-23 mRNA and protein levels at these sites were significantly increased in *Mcpt8^cre/+^ Il4^fl/–^13^fl/+^* mice compared with controls (Figure 4, M–O). In addition, *Mcpt8^cre/+^ Il4^fl/–^13^fl/+^* mice exhibited increased cutaneous IL-17A production, due to the increased percentage of IL-17A^+^TCRγδ^+^ cells infiltrating their infected skin (Figure 4P). Importantly, the numbers of *S*. *aureus* CFU recovered from skin homogenates after *S*. *aureus* application to tape-stripped skin were significantly reduced in *Mcpt8^cre/+^ Il4^fl/–^13^fl/+^* mice compared with controls (Figure 4Q). Together, these results indicate that IL-4 derived from basophils recruited to mechanically injured skin promotes cutaneous infection with *S*. *aureus*.

### IL-4 acts at multiple checkpoints to inhibit the protective cutaneous IL-17A response against superficial infection by S. aureus.

**Figure 5:**
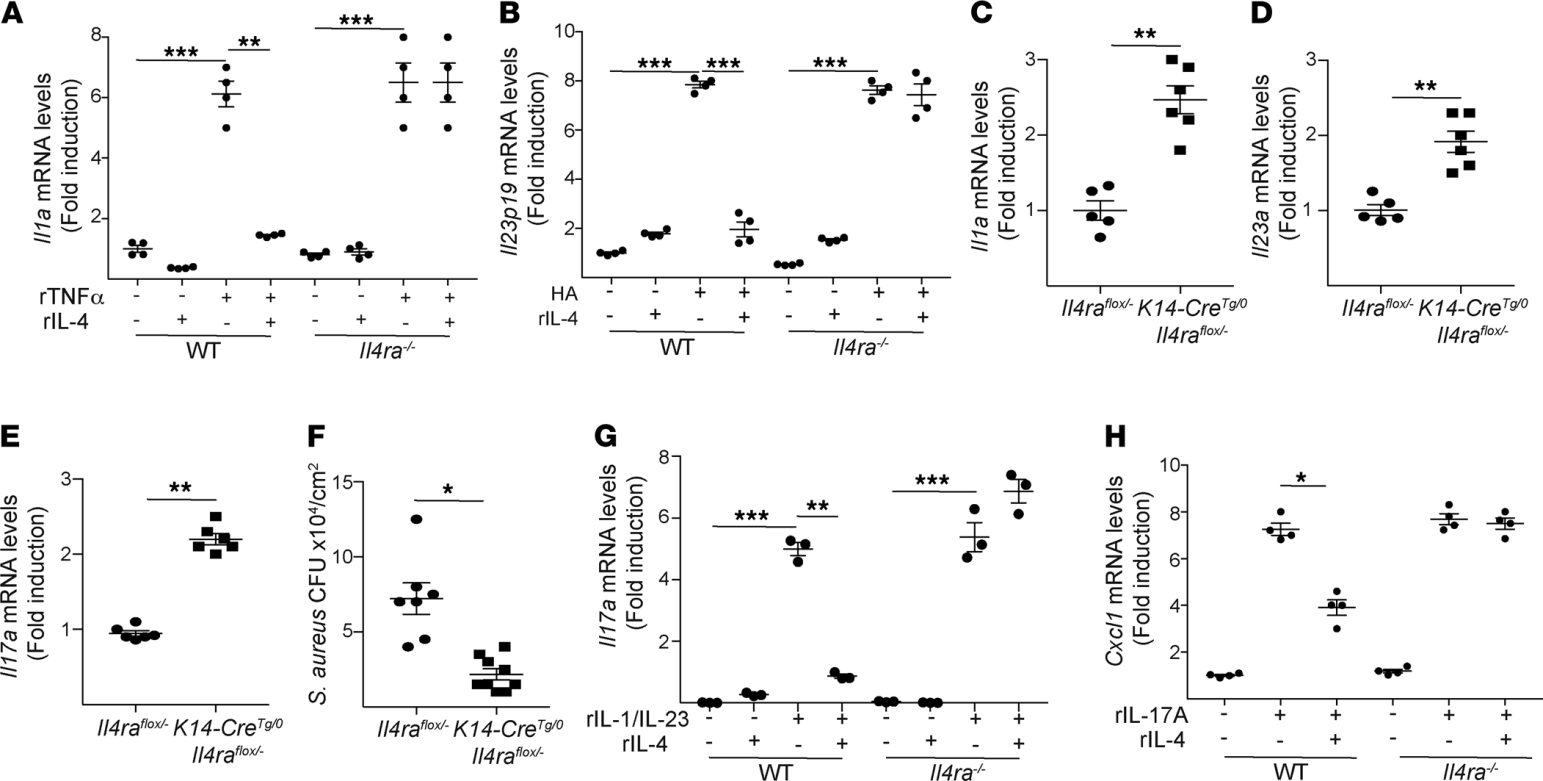
IL-4 acts at multiple checkpoints to inhibit the protective cutaneous IL-17A response against superficial infection by *S. aureus*. (**A** and **B**) Effect of rIL-4 on the induction of *Il1a* by TNF-α (**A**) and *Il23p19* by HA (**B**) in epidermal sheets from *Il4ra^–/–^* mice and WT controls. (**C**–**F**) Cutaneous expression of *Il1a* (**C**), *Il23p19* (**D**), and *Il17a* (**E**) at 24 hours and numbers of CFU (**F**) in tape-stripped (T/S) skin of *K14-Cre^Tg/0^* *Il4ra^fl/–^* mice and *Il4ra^fl/–^* controls 72 hours after topical application of 1 × 10^8^ *S*. *aureus*. (**G**) Effect of IL-4 on IL-1β+IL-23–driven induction of *Il17a* expression in TCRγδ^+^ cells from *Il4ra^–/–^* mice and WT controls. (**H**) Effect of IL-4 on IL-17A–driven induction of *Cxcl1* expression in epidermal sheets from *Il4ra^–/–^* mice and WT controls. Results in **A**–**H** are representative of 2 independent experiments with 4–5 mice/group. *P < 0.05, **P < 0.005, ***P < 0.001 by 1-way ANOVA (**A**, **B**, **G**, and **H**) or 1-tailed Student’s *t* test (**C**–**F**).

Mechanical injury induced by tape stripping promotes the production of damage-associated molecular patterns and cytokines. These include TNF-α and hyaluronic acid (HA), which drive the expression of IL-1α and IL-23, respectively, in keratinocytes (33, 40). Keratinocytes express type II IL-4 receptor (IL-4R). The type II IL-4R binds both IL-4 and IL-13 and shares the IL-4Rα chain with the type I IL-4R, which binds IL-4 but not IL-13. Addition of rIL-4 inhibited TNF-α–driven *Il1a* expression as well as HA-driven *Il23p19* expression by epidermal sheets from WT mice but not *Ilr4a^–/–^* mice (Figure 5, A and B). These in vitro findings suggested that IL-4 could inhibit cutaneous *Il17a* expression in response to *S*. *aureus* exposure by downregulating IL-1α and IL-23 production by keratinocytes. To test this hypothesis, we generated *K14-Cre^Tg/0^* *Il4ra^fl/–^* mice, which lack IL-4Rα selectively in keratinocytes. Cutaneous expression of *Il1a*, *Il23p19*, and *Il17a* following *S*. *aureus* application to tape-stripped skin was significantly increased in *K14-Cre^Tg/0^* *Il4ra^fl/–^* mice compared with *K14-Il4ra^fl/–^* controls (Figure 5, C–E). Importantly, the numbers of CFU recovered from skin homogenates after *S*. *aureus* application to tape-stripped skin were significantly reduced in *K14-Cre^Tg/0^* *Il4ra^fl/–^* mice compared with controls (Figure 5F).

TCRγδ^+^ cells, the major producers of IL-17A in mechanically injured skin exposed to *S*. *aureus*, express type I IL-4R (41). We investigated whether IL-4 has a direct effect on *Il17a* expression by TCRγδ^+^ cells following stimulation with IL-1 and IL-23. rIL-4 markedly inhibited IL-1β+IL-23–driven *Il17a* expression by TCRγδ^+^ cells sorted from the skin of WT mice but not *Ilr4a^–/–^* mice (Figure 5G).

Keratinocytes are a major target for IL-17A–driven expression of neutrophil-attracting chemokines (42). We examined whether IL-4 has a direct effect on IL-17A–driven expression of the neutrophil chemoattractant *Cxcl1* by keratinocytes. rIL-4 inhibited IL-17A–driven expression of the neutrophil-attracting chemokine *Cxcl1* by epidermal sheets from WT mice but not *Ilr4a^–/–^* mice (Figure 5H). The above results suggest that IL-4 may act at multiple checkpoints to inhibit the protective cutaneous IL-17A response of mechanically injured skin to *S*. *aureus* and consequently promote infection.

### IL-4Rα blockade protects mechanically injured skin from superficial S. aureus infection.

**Figure 6:**
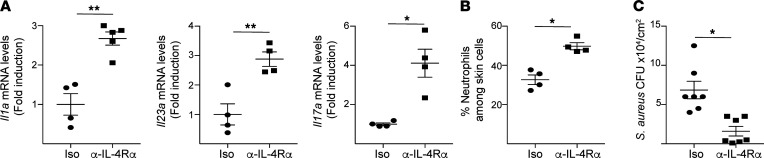
IL-4R blockade protects mechanically injured skin from superficial *S. aureus* infection. (**A**–**C**) Cutaneous expression of *Il1a*, *Il23p19*, and *Il17*a (**A**); neutrophil infiltration in the skin (**B**) at 24 hours; and numbers of CFU (**C**) 72 hours after topical application of 1 × 10^8^ *S*. *aureus* strain to tape-stripped (T/S) skin of WT recipients of anti–IL-4Rα antibody or IgG isotype control. Results in **A**–**C** are representative of 2 independent experiments with 4–5 mice/group. *P < 0.05, **P < 0.005 by 1-tailed Student’s *t* test.

IL-4Rα blockade by mAb is approved for human use. We investigated whether IL-4Rα blockade could protect against *S*. *aureus* infection of mechanically injured skin. Mice were administered rat anti–mouse IL-4Rα–blocking mAb or IgG isotype control 2 hours prior to application of *S*. *aureus* to tape-stripped skin. Cutaneous expression of *Il1a*, *Il23p19*, and *Il17a* as well as neutrophil infiltration were significantly higher in *S*. *aureus*–exposed, tape-stripped skin of recipients of the IL-4Rα–blocking antibody compared with recipients of IgG isotype control (Figure 6, A and B). However, there were no significant changes in *Il4* mRNA levels or in the percentages of TCRγδ^+^ cells and basophils following IL-4Rα antibody blockade (Supplemental Figure 3). Importantly, IL-4Rα blockade significantly reduced the numbers of *S*. *aureus* CFU recovered from the skin (Figure 6C). These results indicate that IL-4 signaling blockade restrains *S*. *aureus* infection of mechanically injured skin. 

## Discussion

We have uncovered an important role for basophil-derived IL-4 in promoting superficial skin infection with *S*. *aureus*. Our results demonstrate that basophils are recruited following exposure of *S*. *aureus* on tape-stripped skin and that basophil-derived IL-4 acts at multiple checkpoints to inhibit the IL-17A response of TCRγδ^+^ T cells that protects against infection. Importantly, we show that IL-4Rα blockade, currently in use for the treatment of patients with AD, protects tape-stripped skin from superficial *S*. *aureus* infection.

Shaved but otherwise intact mouse skin was resistant to the establishment of superficial skin infection by *S*. *aureus*. In contrast, mouse skin mechanically injured by tape stripping was permissive for *S*. *aureus* superficial skin infection. Like intradermal and subcutaneous infection with *S*. *aureus*, *s*uperficial *S*. *aureus* skin infection elicited a TCRγδ^+^ cell–dependent protective local IL-17A response that promoted neutrophil recruitment and AMP production, both of which are important for the clearance of *S*. *aureus* (7, 8, 14, 43). IL-1 and IL-23, which may be derived in part from keratinocytes, were required for the induction of IL-17A following *S*. *aureus* infection of mechanically injured skin, consistent with the important role of these 2 cytokines in the induction of IL-17A in inflammation, infections, and wound healing (16, 18, 19, 34). IL-1 signaling was required for the optimal induction of cutaneous *Il23p19* expression in skin superficially infected with *S*. *aureus*. This is in line with a recent report indicating that recombinant IL-1R antagonist decreases *Il23p19* expression and neutrophil recruitment into the skin induced by ablation of corneodesmosin (44).

We have shown that basophils are recruited to mechanically injured skin exposed to *S*. *aureus* and partially block the IL-17A/neutrophil recruitment axis that protects against superficial skin infection. In agreement with this result, acute depletion of basophils exacerbates mouse models of experimental autoimmune encephalomyelitis (EAE) and colitis mediated by IL-17A and neutrophils (26, 28, 45). Other studies have shown that basophils promote type 17 immune responses by producing IL-6 and that chronic depletion of basophils exacerbates the lupus phenotype in MRL-*lpr/lpr* mice and the severity of EAE in WT mice (25, 27). Thus, basophils may play a context-dependent dual role in the in vivo IL-17 response, initially suppressing its induction, then once it is established, supporting its maintenance.

Basophils are a key source of IL-4 under steady-state as well as pathological conditions (38). Our results demonstrate that basophils are the main source of IL-4 in skin superficially infected with *S*. *aureus* and that basophil-derived IL-4 dampens the protective IL-17A response against infection. This inhibition is in line with the known role of IL-4 in suppressing in type 17 responses. IL-4 suppresses IL-17A expression by CD4^+^ T cells as well as the in vitro production of the IL-17–inducing cytokines IL-23p19, IL-1β, and IL-6 by DCs and keratinocytes (24, 36). In vivo, treatment with rIL-4 improves disease severity in mouse models of EAE and psoriasis, 2 disease models associated with a type 17 immune response (36, 46, 47).

Keratinocytes have emerged as important players in cutaneous immune responses, as they respond to autocrine and paracrine cytokines by producing cytokines, chemokines, and AMP. We show that IL-4 suppresses *Il1a*, *Il23p19*, and *Cxcl1* expression by epidermal sheets in response to in vitro stimulation with TNF-α, HA, and IL-17A, respectively. Importantly, mice lacking IL-4Rα selectively in keratinocytes demonstrated increased cutaneous expression of the cytokines *Il1a*, *Il23p19*, and *Il17a* in mechanically injured skin exposed to *S*. *aureus*. In line with these results, IL-4 inhibits TNF-α– as well as IFN-γ–driven expression of AMP-encoding genes by keratinocytes (48). It also suppresses the production of IL-1β and the AMP hBD2 in epidermal cells from patients with psoriasis (49), who exhibit increased IL-4Rα expression by epidermal cells (50). Together, these results suggest that IL-4 signaling in keratinocytes inhibits cutaneous IL-17A induction and plays a counterpoint to IL-17 signaling in these cells, possibly in an attempt to restore homeostasis in inflamed skin.

The diversity of the skin microbiome and its interactions with pathogenic microorganisms shape the immune response and limit the growth of pathogens (51–53). We cannot exclude that perturbation of skin microbiome induced by tape stripping or in mice with deficiencies in cells or cytokines could affect the establishment of cutaneous *S*. *aureus* infection. Furthermore, different *S*. *aureus* strains produce different virulence factors and toxins that could alter the protective immune response differently than the *S*. *aureus* SF8300 strain we used.

Administration of dupilumab, a human mAb against IL-4Rα, to patients with AD ameliorates the disease and results in improved local and systemic disease markers, as well as a decrease in *S*. *aureus* colonization of the skin (54–57). It is difficult to dissect the exact mechanisms by which IL-4Rα blockade reduces the *S*. *aureus* cutaneous burden in patients with AD. We demonstrate that IL-4Rα blockade increases *Il17a* expression and promotes bacterial clearance from the skin in our mouse model of acute superficial infection with *S*. *aureus*. This is of clinical relevance because it suggests that IL-4R blockade might represent an effective therapeutic approach to inhibit *S*. *aureus* skin colonization or prevent *S*. *aureus* SSTIs.

## Methods

### Mice.

BALB/c and C57BL/6J WT mice were purchased from Charles River Laboratories. *Rag2*^–/–^ mice were purchased from Taconic. *Il17a*^–/–^ mice were previously described (58). *Tcrd^–/–^*, *Il1r1^–/–^*, *Il4^–/–^*, and *Il4ra^–/–^* mice were purchased from The Jackson Laboratory. *Il4/13^fl/fl^* mice were a gift of Andrew McKenzie (Medical Research Council, Laboratory of Molecular Biology, Cambridge, United Kingdom). *Mcpt8^DTR^* mice were a gift of TMDU and TMDU Advanced Research Institute, Tokyo, Japan. *Il4ra^fl/fl^* mice was a gift from International Center for Genetic Engineering and Biotechnology, University of Cape Town, and South Africa Medical Research Council, Cape Town, South Africa. *Mcpt8^cre/+^* mice on C57BL/6J background were purchased from The Jackson Laboratory and crossed with *Il4^–/–^* mice on BALB/c background for 8 generations, then crossed with *Il4/13^fl/fl^* mice in BALB/c background. *K14-Cre^Tg/0^* mice on C57BL/6J background were purchased from The Jackson Laboratory and crossed with *Il4ra^–/–^* in BALB/c background for 9 generations, then crossed with *Il4ra^fl/fl^* mice in BALB/c background. All mice were kept in a pathogen-free environment.

### Skin cell preparation and flow cytometry.

Skin pieces (1 cm^2^) from unmanipulated, tape-stripped, or *S*. *aureus*–infected mice were obtained. Skin pieces were finely chopped using scissors after fat removal and digested for 90 minutes in media containing liberase (Roche) and DNAse II (MilliporeSigma), with continuous shaking at 37°C. Digested skin homogenates were filtered, washed, and resuspended in PBS and used for flow cytometry. Cells were preincubated with Fcγ receptor–specific blocking mAb (clone 2.4G2, BioLegend) and washed before staining with the following mAbs: Alexa Fluor 700–anti–CD45 (clone 30-F11, BioLegend), BV605–anti-CD11b (clone M1/70, BioLegend), FITC–anti-Gr1 (clone RB6-8C5, BioLegend), CD3–efluor 450 (clone 17A2, eBioscience), IgE-PE (RME-1, BioLegend), and CD117-APC (clone ACK2, eBioscience). Cells were analyzed on LSR Fortessa (BD Biosciences), and the data were analyzed with FlowJo software.

### Quantitative reverse transcription PCR.

At indicated times after tape stripping or *S*. *aureus* infection, total skin and epidermal and dermal sheet RNA was extracted with Total RNA Isolation Kit (Ambion). cDNA was prepared with iScript cDNA Synthesis Kit (Bio-Rad). PCR reactions were run on an ABI Prism 7300 (Applied Biosystems) sequence detection system platform. TaqMan primers and probes were obtained from Life Technologies. The housekeeping gene β_2_-microglobulin was used as an internal control.

### S. aureus and quantification of skin infection.

The community-acquired MRSA USA300 SF8300 strain, a gift of Binh Diep (UCSF, San Francisco, California, USA) and the GFP-producing USA300 LAC strain (59) were cultured in tryptic soy broth as previously described. Briefly, a *S*. *aureus* inoculum was streaked onto a tryptic soy agar plate and grown overnight at 37°C. Single colonies were picked and inoculated into a 5 mL tube containing tryptic soy broth and cultured overnight in a shaking incubator. The following morning, 1:50 dilution of bacterial suspension was inoculated in 5 mL of tryptic soy broth and cultured for another 2 hours. Bacterial concentrations were estimated by measuring absorbance at 600 nm. The bacteria were concentrated to 10^8^ CFU/50 μL of PBS and used for cutaneous infection. CFUs were verified by overnight culturing of inoculum on CHROMagar plates. To enumerate the bacterial load in vivo, *S*. *aureus* was labeled with the PSVue794 reagent kit (LI-COR), following manufacturer’s instructions. PSVue794 fluorescence was quantified at different time points using Pearl Trilogy Small Animal Imaging System (LI-COR). To enumerate the bacterial load from the skin, two 8 mm^2^ skin biopsies were obtained. After mechanical homogenization, serial dilutions of skin homogenates were cultured overnight on CHROMagar plates. The growth of USA300 strain was quantified by counting only pink colonies.

### DTR-mediated cell depletion.

*Mcpt8^DTR^* mice received an i.p. injection of DT (750 ng per 20 g body weight) 2 days before *S*. *aureus* application to tape-stripped skin.

### IL-23 and IL-4Rα blockade.

Mice were injected i.v. with 100 μg of rat monoclonal anti–mouse IL-23p19 antibody (Bio X Cell) or with 100 μg of rat monoclonal anti–mouse IL-4Rα antibody (BD Biosciences) 2 hours before *S*. *aureus* application to tape-stripped skin.

### Preparation of epidermal and dermal sheets and culture.

Ears from mice were split into dorsal and ventral halves, then floated for 30 minutes at 37°C on PBS containing 4 mg/mL dispase (MilliporeSigma). Dermal and epidermal sheets were split and stored at –80°C for RNA analysis. Epidermal sheets were cultured with overnight HA (10 μg/mL), rIL-1 (10 ng/mL), rTNF-α (20 ng/mL), rIL-17A (50 ng/mL), and/or rIL-4 (50 ng/mL).

### Histology.

For H&E and Gram staining, skin specimens were fixed in 4% PFA and embedded in paraffin. Sections (5 μm) of skin were stained with H&E or Gram stained by the Rodent Histopathology Core at Dana-Farber/Harvard Cancer Center. For fluorescence microscopy, 10 μm cryosections were fixed in 4% PFA and permeabilized using BD Biosciences Cytoperm Permeabilization buffer, and then slides were mounted using mounting medium containing DAPI (ProLong Gold) from Thermo Fisher Scientific. Pictures were captured using EVOS cell imaging systems.

### Statistics.

Statistical significance was determined by 1-tailed Student’s *t* test or 1-way ANOVA on GraphPad Prism. All the figures display mean ± SEM. A *P* value less than 0.05 was considered statistically significant.

### Study approval.

All procedures performed on the mice were in accordance with and approved by the Animal Care and Use Committee of Boston Children’s Hospital.

## Author contributions

JMLC and RSG conceived the project and supervised the experiments. JMLC, MD, JK, MS, SS, and DSHW performed, analyzed, and interpreted results of the experiments. ARH, HK, FB, and LSM contributed critical reagents or mice. JMLC and RSG wrote the manuscript.

## Supplementary Material

Supplemental data

## Version 1 11/08/2021

Electronic publication
