# Supplementary material for: Basophil-derived IL-4 promotes cutaneous Staphylococcus aureus infection
Source: JCI Insight. 2021 Nov 8;6(21):e149953. doi: 10.1172/jci.insight.149953 (PMC8663570; doi:10.1172/jci.insight.149953)
Supplement: Supplemental data [file jciinsight-6-149953-s042.pdf]

Supplementary Figure legends.

Figure S1. Effect of topical application of *S aureus* on the cutaneous expression of neutrophil chemoattractants and antimicrobial peptides. A, B. Effect of topical application of *S. aureus* on the expression of neutrophil chemoattractants *Cxcl2* and *Cxcl3* (A) and antimicrobial peptides *Defb1*, *Defb3*, *Defb4*, and *Defb14* (B) in shaved skin and shaved and tape stripped (T/S) skin of BALB/c mice. **C.** Effect of topical application of *S. aureus* on the expression of neutrophil chemoattractants *Cxcl2* and *Cxcl3* and of antimicrobial peptides *Defb4*, and *Defb14* in shaved and T/S skin of $Il17^{-/-}$ mice and WT controls. Results are representative of 2 independent experiments with 4-5 mice/group. ** $p < 0.005$, *** $p < 0.001$.

Figure S2. Flow cytometry gating strategy and quantitation of IL-17A producing TCR $\gamma\delta^+$ cells, $CD4^+$ T cells and ILC3s in the skin of *S. aureus* infected mice. A, B. Representative (left) and quantitative (right) flow cytometry analysis of IL-17A producing TCR $\gamma\delta^+$ cells ($CD45^+CD3^+TCR\ \gamma^+$), $CD4^+$ T cells ($CD45^+CD3^+CD4^+$), and ILC3s ($CD45^+CD3^-$ Lin^- $CD90^+Rorgc^+$) in shaved and tape stripped (T/S) skin of Balb/c mice 24 h after application of *S. aureus*. Results are representative of 1 experiment with 5 mice/group.

Figure S3. IL-4R blockade does not affect cutaneous *Il4* mRNA levels or percentages of basophils and TCR $\gamma\delta^+$ cells in the skin of *S. aureus* infected mice. A-B. Cutaneous expression of *Il4* (A) and percentages of basophils and TCR $\gamma\delta^+$ cells 24 h after topical applicationof $1x10^8$ *S. aureus* to tape stripped skin of WT recipients of anti-IL-4Rα antibody or IgG isotype control. Results in A-C are representative of 2 independent experiments with 4-5mice/group.

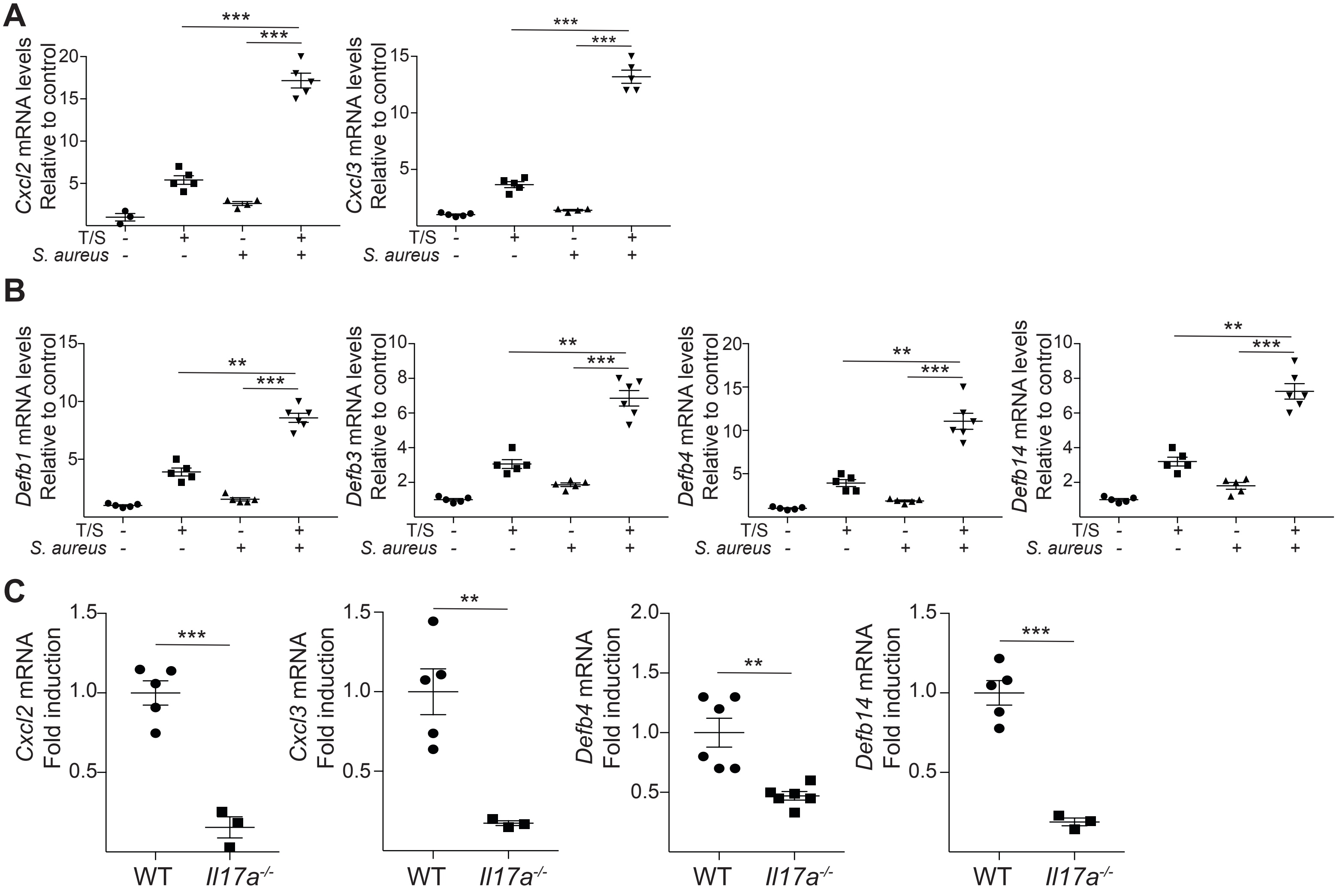
A
Cxcl2 mRNA levels
Relative to control
Cxcl3 mRNA levels
Relative to control
T/S
S. aureus
***
B
Defb1 mRNA levels
Relative to control
Defb3 mRNA levels
Relative to control
Defb4 mRNA levels
Relative to control
Defb14 mRNA levels
Relative to control
**
C
Cxcl2 mRNA
Fold induction
Cxcl3 mRNA
Fold induction
Defb4 mRNA
Fold induction
Defb14 mRNA
Fold induction
WT
Il17a-/-

Figure S1

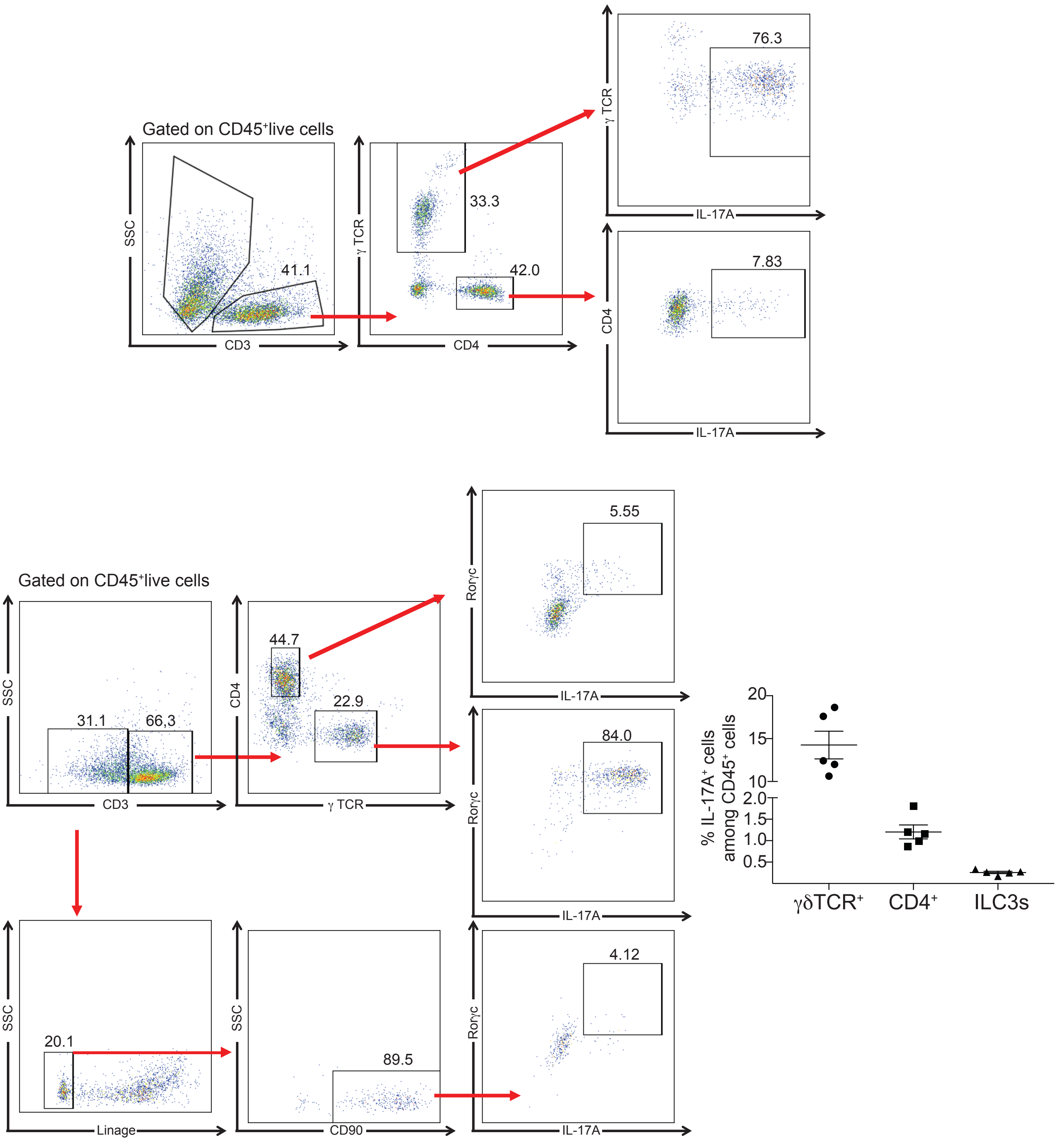

Gated on CD45⁺live cells
SSC
CD3
41.1
γ TCR
CD4
33.3
42.0
γ TCR
IL-17A
76.3
CD4
IL-17A
7.83
Gated on CD45⁺live cells
SSC
CD3
31.1
66,3
CD4
γ TCR
44.7
22.9
Rorγc
IL-17A
5.55
Rorγc
IL-17A
84.0
SSC
Linage
20.1
SSC
CD90
89.5
Rorγc
IL-17A
4.12
% IL-17A⁺ cells among CD45⁺ cells
20
15
10
2.0
1.5
1.0
0.5
γδTCR⁺
CD4⁺
ILC3s

Figure S2

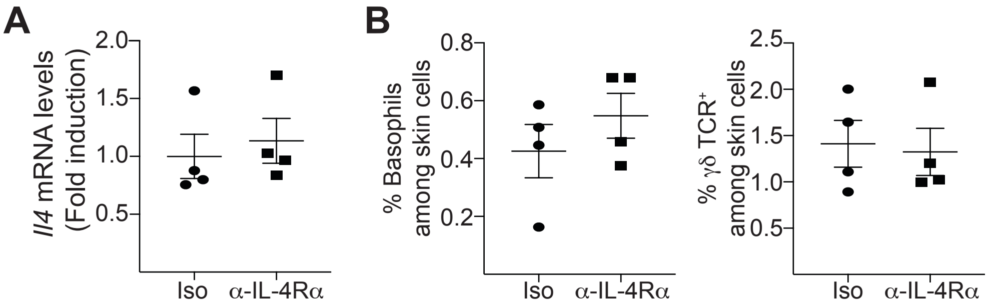

A
B
Il4 mRNA levels
(Fold induction)
2.0
1.5
1.0
0.5
Iso
α-IL-4Rα
% Basophils
among skin cells
0.8
0.6
0.4
0.2
Iso
α-IL-4Rα
% γδ TCR+
among skin cells
2.5
2.0
1.5
1.0
0.5
Iso
α-IL-4Rα

Figure S3
